# Management of Localized Melanoma in the Anti-PD-1 Era

**DOI:** 10.1007/s11912-024-01556-z

**Published:** 2024-06-06

**Authors:** Elan Novis, Alexander C. J. van Akkooi

**Affiliations:** 1 Melanoma Institute Australia, 40 Rocklands Road, Wollstonecraft, North Sydney, Sydney, NSW 2060 Australia; 2 Faculty of Medicine and Health, University of Sydney, Sydney, NSW Australia; 3 Department of Melanoma and Surgical Oncology, Royal Prince Alfred Hospital, Sydney, NSW Australia

**Keywords:** Cutaneous melanoma, Immunotherapy, Targeted therapy, Neoadjuvant, Adjuvant, Sentinel Node, Lymph Node

## Abstract

**Purpose of Review:**

The management of cutaneous melanoma has rapidly progressed over the past decade following the introduction of effective systemic therapies. Given the large number of recent clinical trials which have dramatically altered the management of these patients, an updated review of the current evidence regarding the management of localized melanoma is needed.

**Recent Findings:**

The role of effective systemic therapies in earlier stages (I-III) melanoma, both in adjuvant and neoadjuvant settings is rapidly changing the role of surgery in the management cutaneous melanoma, particularly regarding surgical safety margins for wide local excision (WLE), the role of sentinel lymph node biopsy (SLNB) and the extent of lymph node dissections. The randomized phase 2 SWOG1801 trial has demonstrated superiority of neoadjuvant-adjuvant anti-PD1 therapy in improving event-free survival by 23% at 2-years over adjuvant anti-PD-1 therapy only. Furthermore, the PRADO trial has suggested a more tailored approach both the extent of surgery as well as adjuvant therapy can safely and effectively be done, depending on the response to initial neoadjuvant immunotherapy. These results await validation and it is expected that in 2024 the phase 3 Nadina trial (NCT04949113) will definitively establish neo-adjuvant combination immunotherapy as the novel standard. This will further redefine the management of localized melanoma.

**Summary:**

The use of effective systemic therapies will continue to evolve in the next decade and, together with new emerging diagnostic and surveillance techniques, will likely reduce the extent of routine surgery for stage I-III melanoma.

## Background & Introduction

The management of cutaneous melanoma has rapidly progressed over the past decade following the introduction of effective systemic therapies [1–5]. Despite this, the worldwide burden of disease remains high, particularly in populations with fair-skinned individuals of European descent and is expected to rise over the coming decade [6]. Therefore, the appropriate integration of these novel therapies into current treatment algorithms is crucial to improve survival outcomes for melanoma patients.

The role of effective systemic therapies such as PD-1 immune checkpoint blockade (ICB) immunotherapy and BRAF directed targeted therapy (TT) is now well established, both in unresectable stage III and metastatic stage IV (together also known as ‘advanced’) melanoma, as well as in the adjuvant treatment of stage III melanoma with a reduced risk of recurrence and improved distant metastasis-free survival [7–9]. However, the role of these agents in earlier stages (I-II) melanoma, both in adjuvant and neoadjuvant settings is still developing. The initial success of systemic therapies has also prompted debate about the changing role of surgery in the management cutaneous melanoma, particularly regarding surgical margins, lymph node staging and the role of nodal dissection [10, 11]. This has led to a paradigm shift in management from radical surgery with wide margins and extensive lymphadenectomy, to a more multi-disciplinary approach with narrower margins and de-escalated nodal surgery when combined with systemic therapies, to minimise morbidity and improve disease outcomes.

This review aims to discuss the current management of early (stage I-II) and regionally metastatic (stage III) cutaneous melanoma in the era of effective systemic therapy and to discuss future trends in this evolving field.

## Early Melanoma (Stage I/II) – Role of Wide Local Excision (WLE)

Wide-local excision (WLE) is the standard of care in early (Stage I/II) melanoma in order to achieve good local control and prevent disease recurrence. Guidelines around resection margins have evolved from generous 5cm margins in the early 1900s [12], to narrower 2cm margins in thick melanomas and 1-2cm margins in thin and intermediate melanomas, although current guidelines differ worldwide [13].

To date there have been 6 randomised control trials (RCTs) comparing wide versus narrow surgical margins in cutaneous melanoma [14–19]. An initial meta-analysis of these studies found narrow margins (1-2cm) were associated with a worse melanoma-specific survival RR 1.17 (CI 1.03–1.34; p = 0.02) but no difference in local recurrence, RR 1.10 (CI 0.96–1.26; p = 0.2) or overall survival, RR 1.09 (95% CI 0.98–1.22; p = 0.1 [20]. Given the low incidence of local recurrences in early melanoma (0 – 4.2%) and concerns regarding the morbidity of wide excisions, updated meta-analysis of further follow-up studies and new data from the ongoing MELMART II trial found no difference between narrow and wide margins in terms of local recurrence melanoma-specific survival or overall survival. [13]. All of these trials were conducted prior to the availability of effective systemic therapy, and many were also conducted prior to sentinel lymph node biopsy (SLNB) being standard of care in intermediate and thick melanomas. Therefore, the applicability of this evidence in the setting of routine SLNB and effective systemic therapy remains unclear.

Local recurrence rates in early melanoma are relatively low, with rates of around 1–2% for T2-T3 primaries (1–4 mm Breslow thickness) to 3.4–7.6% for T4 primaries (> 4 mm Breslow thickness) when a 2cm margin is achieved [16, 19]. Guidelines vary worldwide regarding optimal margins for intermediate thickness melanoma (1-4mm) [21–25]. However, the definition of local recurrence in the current studies is not homogenous with some defining a recurrence occurring < 5cm from the primary site and others defining < 2cm from the primary site as a local recurrence. In fact, local recurrences are rarely residual melanoma cells that have progressed but are most frequently microscopic satellite/in-transit lesions that were originally a distance of x millimetres away from the primary tumour that have been pulled towards the scar after WLE.

The MelMarT-II trial (NCT03860883), an international multicentre phase 3 RCT comparing 1cm vs 2cm margins in intermediate and high-risk cutaneous melanomas (T2b-T4b) is currently undergoing recruitment and aims to demonstrate non-inferiority of a narrower margin in terms of disease-free survival, overall survival and risk of melanoma recurrence for patients treated with either a 1 or 2 cm WLE [26]. Early results from this pilot phase of this trial have demonstrated a significantly higher rate of reconstruction in the 2-cm arm compared to the 1-cm arm (39.4 vs. 13.6%, respectively; *p* < 0.0001) and associated complication rate, but interestingly no difference in QOL measures between the two groups. Long-term recurrence and survival outcomes from this study are eagerly awaited.

A novel concept altogether is to completely forego a WLE with 1 or 2 cm safety margins in case of clear margins on the initial excision. The justification for WLE is that by taking an extra margin of healthy skin, potential microsatellites which could result in local recurrence are removed [27]. However, this is not supported by any high-quality evidence. Patients without (micro-)satellites cannot benefit from a WLE. Furthermore, the majority of patients that do harbor (micro-)satellites, already have a positive sentinel node as part of the metastatic biology of their disease, which means they require consideration of adjuvant systemic therapy rather than additional surgery. The few cases that do not have an involved SN initially but recur with satellite/in-transit disease may be dealt with through resection and (neo-)adjuvant therapy at that later time rather than subjecting all stage I/II melanoma patients to an immediate WLE at the time of diagnosis. Further prospective studies are needed to determine if this 2-stage approach is still appropriate in the current era.

## Developing Role of Sentinel Lymph Node Biopsy (SLNB)

In addition to excision of the primary melanoma lesion, accurate staging of the draining lymph nodes is required. Identification of lymph node metastases was revolutionized by the sentinel lymph node biopsy (SLNB) technique, first described in 1992 by the late Dr. Donald Morton [28]. SLNB is now the standard of care in melanoma staging, with SLN status established as an important prognostic factor for clinically node-negative melanoma (≥ pT2a) [29, 30]. Current guidelines recommend SLNB for patients with a primary tumor greater than 1.0-mm thick, or thinner melanomas with high-risk features, such as ulceration or increased mitotic rate of ≥ 2/mm2, lymphovascular invasion, or a positive deep biopsy margin, particularly in younger patients [21–25]. In general, if the risk of a histologically positive SLN is greater than 5–10%, most clinicians would consider or recommend a SLNB [31, 32].

There have been three landmark RCTs that have informed our current practice of SLNB. The first Multicenter Selective Lymphadenectomy Trial (MSLT-1) compared patients who underwent WLE with SLNB or WLE with observation, in patients with intermediate thickness melanoma (1.2 to 3.5 mm). In this trial there was a significantly higher 5-year DFS rate in the SLNB group (78.3% vs 73.1%, respectively, p = 0.009), with similar 5-year MSS rates (87.1% vs 86.6%, respectively, p = 0.58). However, the study also found that in the cohort of patients with lymph node metastases, patients who underwent SLNB with CLND had improved 5-year survival compared to those who had lymph node dissection after clinical recurrence (72.3% vs 52.4%, respectively, p = 0.004). This subgroup analysis has been heavily criticised as it did not take into consideration false negative and false positive SLNB [33–35]. This demonstrated the clinical importance of SLNB in both prognostication as well as preventing the morbidity of a potentially unnecessary Elective Lymph Node Dissection (ELND) in patients who were clinically node negative.

Given the morbidity associated with CLND, two further multicenter RCTs were conducted to examine the question of whether CLND was necessary for patients with SLN metastases. The DeCOG-SLT trial [36] and the larger MSLT-2 trial [37] demonstrated that CLND had no significant improvement in MSS compared to observation alone in patients with a positive SLNB. There was a small but significant improvement in DFS in the MSLT-2 CLND group, largely due to a reduction in regional nodal recurrence (HR 0.31; 95% CI 0.24–0.41; *p* = 0.001). There was no difference in DMFS or overall survival in the two studies. Further follow-up studies after MSLT-2 suggest that regional lymph node recurrence can be salvaged in patients who were initially SLN positive and underwent adjuvant systemic therapy and observation [37–39].

These three RCTs have informed current guidelines regarding SLNB, which is now the standard of care in staging of localized early (stage I-II) cutaneous melanoma. However, they were performed prior to the era of effective systemic therapies.

Additionally, all current highly effective systemic therapies were evaluated in clinical trials that required CLND and most required either Stage IIIB or greater, or Stage IIIA with deposits of at least 1 mm in diameter of SLN metastases. In the MSLT-2 and DeCOG-SLT trials only one third of patients had lymph node metastases greater than 1mm. However, MSLT-2 subgroup analyses did not indicate a benefit for CLND in any of the subgroups. If anything, they seemed to indicate the contrary, that patients with larger metastases benefited more from observation rather than CLND. Therefore, in theory, from a scientific point of view, further prospective trials evaluating the role of SLNB and adjuvant treatment without CLND in the era of effective systemic therapies are needed, but the question is if these will ever be conducted. A negative trial (Checkmate 915) looking at combination ipilimumab at a low dose (1 mg/kg) and only once every 6 weeks + nivolumab (3 mg/kg) was the first to no longer require CLND for SLNB positive disease. It did not affect the outcome of patients within the trial [40].

**Fig. 1:**
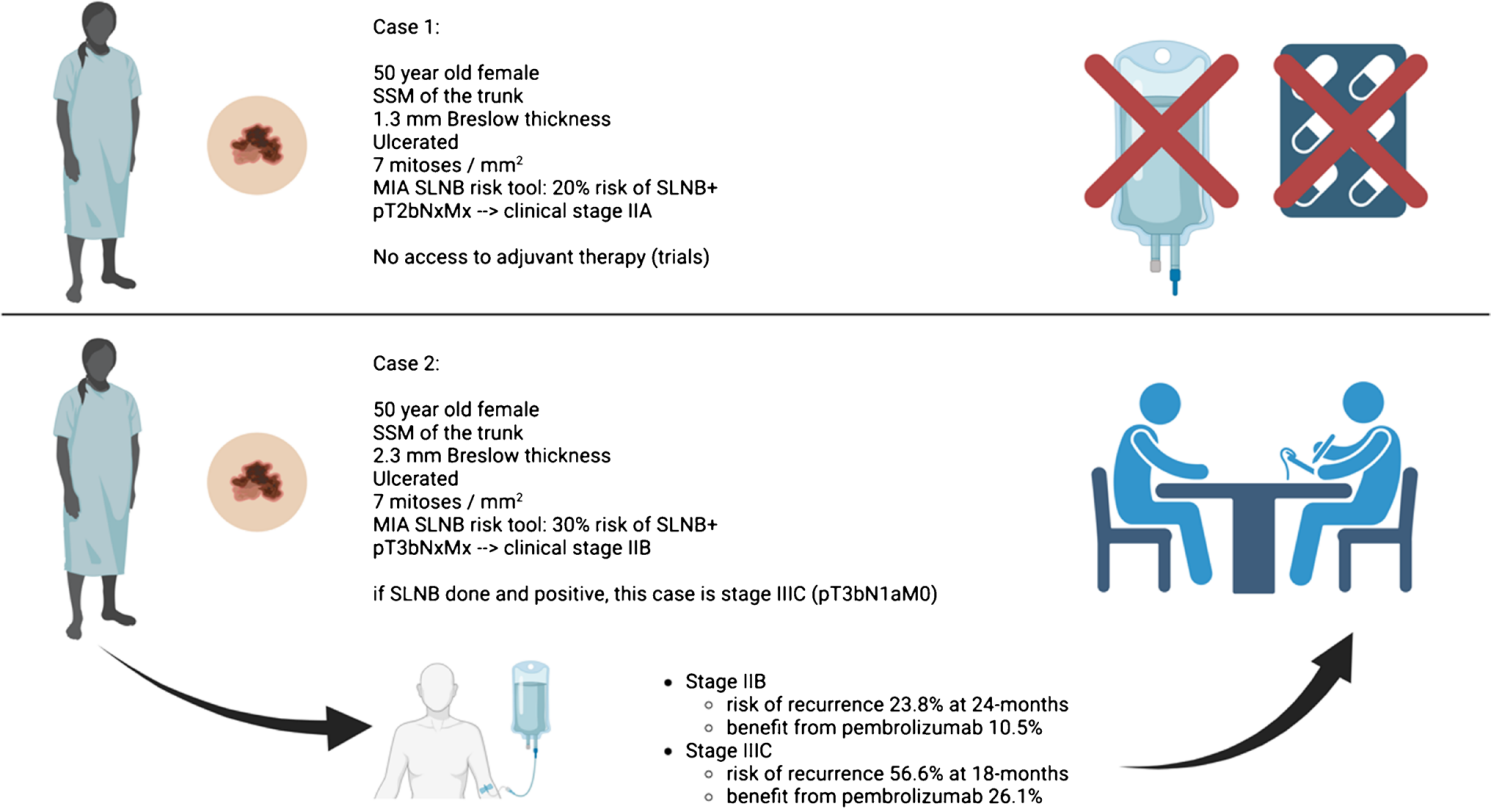
Two case studies demonstrating the importance of SLNB in calculating prognosis and determining benefit from adjuvant systemic therapy

There have been studies that have demonstrated that adjuvant systemic therapies are effective in stage IIB/C melanoma patients too[41–43]. And, therefore one could question the need for SLNB in patients who would be eligible to receive adjuvant therapy, regardless of the outcome of the SLNB? The contrary argument which supports the continued current practice of SLNB in these patients, is that SLNB still provides prognostic information, which helps patients to decide if they wish to proceed with potentially toxic and expensive immunotherapy treatment. SLNB also improves regional disease control, particularly in patients who have adjuvant therapy following SLNB, compared to patient who have adjuvant immunotherapy without SLNB [44, 45]. While patients with thick primary tumors may no longer require SLNB to provide access to adjuvant systemic therapies, the SLNB remains useful in estimating prognosis and reducing the risk of regional nodal recurrence, therefore avoiding the morbidity of a CLND. Figure 1 illustrates a case study and how SLNB is still of importance.

There are some emerging technologies and biomarkers which may increase the tools available to predict the risk of lymph node metastases and therefore reduce the need for SLNB in the future. Biomarkers such as gene expression profilers (GEP), immunohistological signatures and liquid biopsies with ctDNA have the potential to make SLNB obsolete [46]. Ongoing trials such as NivoMela (NCT04309409) a Phase III RCT using biomarker risk stratification to determine which stage II melanomas with a negative SLNB are high risk of recurrence then randomising to either adjuvant nivolumab to observation groups [46]. This could strengthen the argument to forgo SLNB if biomarker analysis alone can stratify patients as high risk, allowing access to adjuvant therapy. ctDNA has also shown promise in stratifying high risk melanomas which may benefit from systemic therapies [47]. In this study ctDNA detection predicted patients at high risk of relapse (HR 2.9; p = 0.002) and was an independent predictor of RFS and DMFS. There is currently, however insufficient evidence to support their use in standard clinical practice.

## Clinical Stage III Melanoma: The Role of Lymph Node Dissection (LND)

Historically, first Elective Lymph Node Dissections (ELND) had been proposed to prophylactically remove potential metastatic spread to the regional draining lymph node(s). Other argued that this approach was too morbid and did not improve survival, therefore a therapeutic lymph node dissection (TLND) at the time of a proven recurrence would be sufficient. It was a pivotal trial by Umberto Veronesi that randomized patients between an ELND or TLND that found no survival benefit for the prophylactic ELND [48].

Later CLND was proposed to have a targeted node dissection only for those patients with a positive SLNB, but as demonstrated above, this has been abandoned after the DECOG-SLT and MSLT-2 trials [36, 37]. Therefore, the current standard of care has reverted to performing a TLND, only in case of a proven macroscopic nodal recurrence (either clinical detected or on imaging). This practice can now, however, be interrogated once again with the coming of the anti-PD-1 era.

All adjuvant systemic therapy trials in macroscopic stage III melanoma have mandated a protocolized TLND before patients were eligible to participate in the trials [7, 9, 40, 49]. Similarly, all neo-adjuvant therapy (NAT) trials have equally mandated a TLND [8, 50, 51••, 52–57]. And finally, all melanoma guidelines [21–25] recommend a TLND for patients with macroscopic nodal involvement. There is no evidence, to our knowledge, to support or reject the practice of performing a ‘node pick’, in which only the involved node on imaging is removed ± adjuvant systemic therapy thereafter. Probably most melanoma surgeons will have experience with single patients that refused TLND, but request this approach. It has, however, not been reported as far as we know.

There might be indirect evidence from the neo-adjuvant, proof-of-concept, PRADO trial. This trial used a NAT regimen of ipilimumab (1 mg/kg) + nivolumab (3 mg/kg) in all patients (n = 99). Patient underwent a resection of the Index Lymph Node (ILN) thereafter. The definition of the ILN was the largest lymph node metastasis at baseline. After resection of the ILN, the subsequent treatment would be a based on the histopathological response in the ILN. This personalized response-driven decision was protocolized as follows: 1) patients with a major pathologic response (MPR = pathologic complete response [pCR] + [near-pCR], maximum of 10% viable tumor cells), would neither have further surgery, nor adjuvant systemic therapy. 2) patients with a pathologic partial response (pPR =  > 10%, < 50% viable tumor cells) would undergo a second surgery to complete a TLND, but received no adjuvant systemic therapy. 3) patients with a pathologic non-response (pNR =  > 50% viable tumor cells) would undergo both a TLND in a second surgery, as well as adjuvant systemic therapy (either switch to BRAF/MEK inhibition for BRAF mutant melanomas or continuation of anti-PD-1) ± adjuvant radiotherapy to the node field [58••]. The trial demonstrated that it was safe and feasible to perform this stepwise treatment approach. The trial showed a 61% MPR rate and in the end, only 30/99 patients underwent a TLND [58••]. The 2-year RFS and DMFS for MRP patients (in the absence of TLND and adjuvant systemic therapy) was 93% and 98%, respectively. MPR patients experience significantly lower surgical morbidity, including wound problems and most importantly, chronic lymphedema of the limb. This translated into an improved health-related quality of life (HRQoL) [58••].

These findings from the PRADO study speak to the imagination of patients and physicians alike, as historically, lymph node dissections, particularly groin dissection have been notorious in terms of morbidity [59]. Especially, chronic lymphedema has been widely recognized to be associated with a worse HRQoL and this avoiding this would be of obvious benefit [60].

## Future

The way we manage localized melanoma has been changing continuously during the last century, from WLE with 5 cm margins to nowadays 1 or 2 cm margins. The management of the draining regional lymph nodes has evolved from ELND to SLNB + CLND to SLNB and TLND in cases of relapse. SLNB is once again being questioned and even TLND might not continue to be the standard of care approach for macroscopic node involvement in the future, if an ILN procedure after NAT becomes the newest standard of care.

First, regarding the WLE procedure, MelMart-2 might further reduce the use of ‘wider’ WLE by establishing the 1 cm margin as the safe margin for all primary cutaneous melanoma patients. However, we propose that in the future, it might be feasible to reduce WLE even further, towards clear margins (1 mm) only as being sufficient. However, this would require a prospective randomized controlled trial in order for the community to accept this practice [61]. It might not be suitable for all melanoma subtypes, such as for example acral or lentiginous melanomas, where it is not uncommon to have a large area of in situ ‘field effect’ present in the area adjacent to the invasive melanoma. However, for the majority of primary melanoma cases, that are the superficial spreading (SSM)(~ 60–70%) or the nodular (NM)(~ 10–15%) subtypes, this approach might be safe and feasible to help further reduce morbidity, improve HRQoL and reduce health care costs as in the USA only nearly 100.000 new cases of melanoma are diagnosed annually (85% stage I/II). Although the morbidity of a 1 cm WLE is low on an individual basis, looking at the sheer number of absolute cases per annum globally, this is a large health care problem.

We have not even discussed the 0.5 – 1.0 cm margins that are advised for in situ melanomas, which have even less evidence to support than the 1 or 2 cm margins from invasive melanomas. Considering the very low rate of local recurrences after WLE or satellite metastases found in a WLE specimen, we argue that in situ melanoma, again with the exception of the acral and lentiginous subtypes, would also benefit from no longer needing to undergo a WLE. The metastatic biology of patients who are destined to progress, recur and/or metastasize cannot be prevented by more aggressive surgery.

**Fig. 2:**
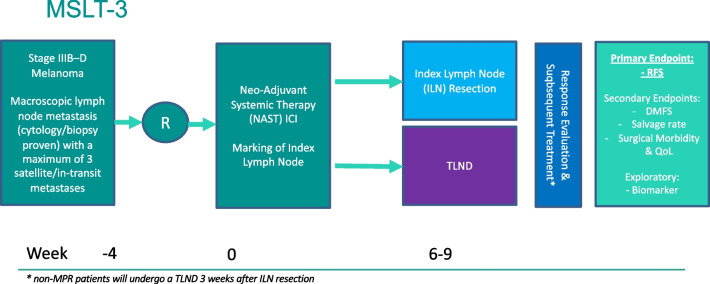
Proposed Phase 3 Prospective RCT to compare ILN resection to TLND following NAST

Second, the reduced extent of therapeutic lymph node dissections, which is already changing of late. PRADO was a proof-of-concept trial and has been received with excitement but has been quoted to be insufficient as a small and single prospective trial to change standard of care practice and guidelines as of yet [62]. This cannot be seen without the establishment of NAT as the novel standard of care for macroscopically involved lymph nodes with melanoma. The SWOG-1801 study was hailed as a potentially practice changing trial [63••], but FDA has not approved pembrolizumab for this indication, because of concerns regarding the statistical power of this phase 2 trial [64–66]. More convincing is the fact that the SWOG-1801 study essentially confirmed the findings from the previous independent investigator-initiated neoadjuvant immune checkpoint inhibitor trials [64–66] and a pooled analysis from the International Neoadjuvant Melanoma Consortium (INMC) [10]. We hope that NAT will be definitively established as the novel standard of care after the Nadina trial (NCT04949113). Regardless, thereafter, the ILN concept needs to be validated in a larger prospective trial as well moving forward. We have proposed a prospective phase 3 randomized controlled trial to address this issue (Fig. 2).

## Conclusions

The management of localized melanoma in the anti-PD-1 era has evolved and will continue to evolve. It is expected that the extent of surgery will be further reduced in order to diminish morbidity, reduce detrimental effects on HRQoL and cut costs to health care systems.
